# 1,4-Bis(2-pyridylmeth­oxy)benzene

**DOI:** 10.1107/S1600536809035946

**Published:** 2009-09-12

**Authors:** Jin-Sheng Gao, Ying Liu, Shuang Zhang, Guang-Feng Hou, Peng-Fei Yan

**Affiliations:** a College of Chemistry and Materials Science, Heilongjiang University, Harbin 150080, People’s Republic of China

## Abstract

In the title compound, C_18_H_16_N_2_O_2_, the phenyl­ene ring is located on inversion center. The pyridyl ring makes a dihedral angle of 39.9 (1)° with the phenyl­ene ring. In the crystal, adjacent mol­ecules are linked by inter­molecular C—H⋯N hydrogen bonds, forming a linear chain along the *a *axis.

## Related literature

For the synthesis of silver and palladium complexes with the 1,4-bis­(2-pyridylmeth­oxy)benzene ligand, see: Hartshorn & Steel (1998 ▶); Oh *et al.* (2005 ▶). For a related structure, see: Gao *et al.* (2006 ▶). For the synthesis of title compound, see: Gao *et al.* (2004 ▶).
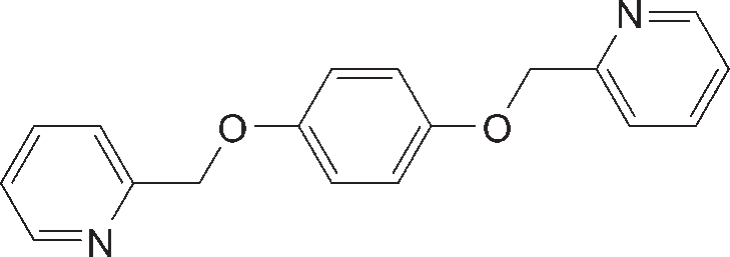


## Experimental

### 

#### Crystal data

C_18_H_16_N_2_O_2_
*M* *_r_* = 292.33
Monoclinic, 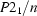

*a* = 9.802 (7) Å
*b* = 3.988 (2) Å
*c* = 18.421 (11) Å
β = 93.77 (3)°
*V* = 718.6 (8) Å^3^
*Z* = 2
Mo *K*α radiation
μ = 0.09 mm^−1^
*T* = 291 K
0.33 × 0.30 × 0.22 mm

#### Data collection

Rigaku RAXIS-RAPID diffractometer
Absorption correction: multi-scan (*ABSCOR*; Higashi, 1995 ▶) *T*_min_ = 0.972, *T*_max_ = 0.980
6515 measured reflections
1639 independent reflections
1308 reflections with *I* > 2σ(*I*)
*R*_int_ = 0.024

#### Refinement

*R*[*F* ^2^ > 2σ(*F* ^2^)] = 0.039
*wR*(*F* ^2^) = 0.118
*S* = 1.11
1639 reflections
100 parameters
H-atom parameters constrained
Δρ_max_ = 0.18 e Å^−3^
Δρ_min_ = −0.18 e Å^−3^

### 

Data collection: *RAPID-AUTO* (Rigaku 1998 ▶); cell refinement: *RAPID-AUTO*; data reduction: *CrystalStructure* (Rigaku/MSC 2002 ▶); program(s) used to solve structure: *SHELXS97* (Sheldrick, 2008 ▶); program(s) used to refine structure: *SHELXL97* (Sheldrick, 2008 ▶); molecular graphics: *SHELXTL* (Sheldrick, 2008 ▶); software used to prepare material for publication: *SHELXL97*.

## Supplementary Material

Crystal structure: contains datablocks I, global. DOI: 10.1107/S1600536809035946/ng2637sup1.cif

Structure factors: contains datablocks I. DOI: 10.1107/S1600536809035946/ng2637Isup2.hkl

Additional supplementary materials:  crystallographic information; 3D view; checkCIF report

## Figures and Tables

**Table 1:** Hydrogen-bond geometry (Å, °)

*D*—H⋯*A*	*D*—H	H⋯*A*	*D*⋯*A*	*D*—H⋯*A*
C9—H9⋯N1^i^	0.93	2.68	3.587 (2)	165

Symmetry code: (i) 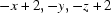.

**Fig. 1.**
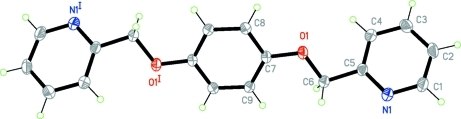
The molecular structure of the title compound, showing displacement ellipsoids at the 30% probability level for non-H atoms.

**Fig. 2.**
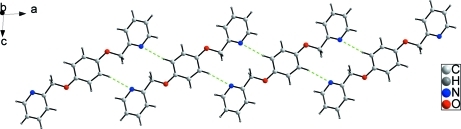
A partial packing view, showing the one-dimensional hydrogen bonding chain. Dashed lines indicate the hydrogen-bonding interactions and no involving H atoms have been omitted.

## supplementary crystallographic information

### Comment

The bipyridyl ligand is generally used as bridge units to construct metal-organic framework. Hartshorn's group have reported the syntheses of the silver and palladium complexes with the 1,4-bis(2-pyridylmethoxy)benzene ligand, which assemble into one-dimensional zigzag chain in the former and an *M*_2_*L*_2_ 26-membered macrocycle in the latter (Hartshorn *et al.*, 1998). Oh's group have investeigated how metal-ligand stoichiometry can be used to influence the formation of polymeric structures, in which they reacted silver salts with the 1,4-bis(2-pyridylmethoxy)benzene ligand in 1:1 ratio to form one-dimensional zigzag chain and in 1:2 ratio to yield a two-dimensional porous network. Herein we synthesized the same ligand and hoped to obtain the fluorescent material by reacting the ligand with d^10^ metal, but we get a lot of crystals of the ligand itself and report its crystal structure here.

The X-ray single-crystal analysis of the title compound shows that the 1,4-bis(2-pyridylmethoxy)benzene molecule is centrosymmetric. The planes of two terminal pyridyl groups are parallel and make dihedral angles of 39.9 (1) ° with the plane of the central benzene ring (Figure 1). In the crystal structure, the 1,4-bis(2-pyridylmethoxy)benzene molecules are linked by intermolecular C—H···N hydrogen bonds into one dimensional chains along *a* axis direction (Table 1, Figure 2).

### Experimental

The 1,4-bis(2-pyridylmethoxy)benzene was synthesized by the reaction of *p*-benzenediol and 2-chloromethylpyridine hydrochloride under nitrogen atmosphere and alkaline condition (Gao *et al.*, 2004; Gao *et al.*, 2006). A solution of Zn(NO_3_)_2_.6H_2_O (0.3 g, 1 mol) in water (5 ml) was dropped slowly into a methanol solution (5 mL) of 1,4-bis(2-pyridylmethoxy)benzene (1.46 g, 5 mmol) to give a clear solution. Colourless nod-shaped crystals of scheme were obtained by slow evaporation of the clear solution after four days.

### Refinement

H atoms bound to C atoms were placed in calculated positions and treated as riding on their parent atoms, with C—H = 0.93 Å (aromatic), C—H = 0.97 Å (methylene), and with *U*_iso_(H) = 1.2*U*_eq_(C).

### Crystal data

**Table:**

C_18_H_16_N_2_O_2_	*F*(000) = 308
*M**_r_* = 292.33	*D*_x_ = 1.351 Mg m^−^^3^
Monoclinic, *P*2_1_/*n*	Mo *K*α radiation, λ = 0.71073 Å
Hall symbol: -P 2yn	Cell parameters from 5105 reflections
*a* = 9.802 (7) Å	θ = 3.3–24.5°
*b* = 3.988 (2) Å	µ = 0.09 mm^−^^1^
*c* = 18.421 (11) Å	*T* = 291 K
β = 93.77 (3)°	Block, colorless
*V* = 718.6 (8) Å^3^	0.33 × 0.30 × 0.22 mm
*Z* = 2	

### Data collection

**Table:**

Rigaku RAXIS-RAPID diffractometer	1639 independent reflections
Radiation source: fine-focus sealed tube	1308 reflections with *I* > 2σ(*I*)
graphite	*R*_int_ = 0.024
ω scan	θ_max_ = 27.5°, θ_min_ = 3.8°
Absorption correction: multi-scan (*ABSCOR*; Higashi, 1995)	*h* = −12→12
*T*_min_ = 0.972, *T*_max_ = 0.980	*k* = −5→4
6515 measured reflections	*l* = −23→23

### Refinement

**Table:**

Refinement on *F*^2^	Primary atom site location: structure-invariant direct methods
Least-squares matrix: full	Secondary atom site location: difference Fourier map
*R*[*F*^2^ > 2σ(*F*^2^)] = 0.039	Hydrogen site location: inferred from neighbouring sites
*wR*(*F*^2^) = 0.118	H-atom parameters constrained
*S* = 1.11	*w* = 1/[σ^2^(*F*_o_^2^) + (0.0651*P*)^2^ + 0.0675*P*] where *P* = (*F*_o_^2^ + 2*F*_c_^2^)/3
1639 reflections	(Δ/σ)_max_ = 0.001
100 parameters	Δρ_max_ = 0.18 e Å^−^^3^
0 restraints	Δρ_min_ = −0.18 e Å^−^^3^

### Special details

**Table:**

Geometry. All e.s.d.'s (except the e.s.d. in the dihedral angle between two l.s. planes) are estimated using the full covariance matrix. The cell e.s.d.'s are taken into account individually in the estimation of e.s.d.'s in distances, angles and torsion angles; correlations between e.s.d.'s in cell parameters are only used when they are defined by crystal symmetry. An approximate (isotropic) treatment of cell e.s.d.'s is used for estimating e.s.d.'s involving l.s. planes.
Refinement. Refinement of *F*^2^ against ALL reflections. The weighted *R*-factor *wR* and goodness of fit *S* are based on *F*^2^, conventional *R*-factors *R* are based on *F*, with *F* set to zero for negative *F*^2^. The threshold expression of *F*^2^ > σ(*F*^2^) is used only for calculating *R*-factors(gt) *etc*. and is not relevant to the choice of reflections for refinement. *R*-factors based on *F*^2^ are statistically about twice as large as those based on *F*, and *R*- factors based on ALL data will be even larger.

### Fractional atomic coordinates and isotropic or equivalent isotropic displacement parameters (Å^2^)

**Table:**

	*x*	*y*	*z*	*U*_iso_*/*U*_eq_	
C1	1.12833 (12)	0.4754 (4)	0.84073 (7)	0.0494 (4)	
H1	1.2127	0.5728	0.8536	0.059*	
C2	1.09220 (13)	0.4307 (4)	0.76808 (7)	0.0478 (4)	
H2	1.1505	0.4972	0.7329	0.057*	
C3	0.96805 (14)	0.2856 (4)	0.74848 (7)	0.0484 (4)	
H3	0.9408	0.2502	0.6998	0.058*	
C4	0.88466 (12)	0.1935 (4)	0.80254 (6)	0.0430 (3)	
H4	0.7999	0.0958	0.7908	0.052*	
C5	0.92844 (11)	0.2482 (3)	0.87452 (6)	0.0339 (3)	
C6	0.84341 (11)	0.1455 (3)	0.93572 (6)	0.0381 (3)	
H6A	0.8602	−0.0879	0.9481	0.046*	
H6B	0.8667	0.2812	0.9785	0.046*	
C7	0.60652 (11)	0.0920 (3)	0.95792 (6)	0.0360 (3)	
C8	0.47231 (12)	0.1610 (3)	0.93481 (6)	0.0393 (3)	
H8	0.4536	0.2702	0.8907	0.047*	
C9	0.63449 (11)	−0.0704 (3)	1.02374 (6)	0.0388 (3)	
H9	0.7243	−0.1181	1.0399	0.047*	
N1	1.04938 (10)	0.3872 (3)	0.89418 (5)	0.0442 (3)	
O1	0.70397 (8)	0.1927 (3)	0.91212 (4)	0.0492 (3)	

### Atomic displacement parameters (Å^2^)

**Table:**

	*U*^11^	*U*^22^	*U*^33^	*U*^12^	*U*^13^	*U*^23^
C1	0.0300 (6)	0.0620 (9)	0.0568 (8)	−0.0047 (6)	0.0072 (5)	0.0111 (7)
C2	0.0370 (6)	0.0591 (9)	0.0492 (7)	0.0080 (6)	0.0182 (5)	0.0146 (6)
C3	0.0462 (7)	0.0630 (9)	0.0368 (6)	0.0055 (6)	0.0087 (5)	0.0019 (6)
C4	0.0337 (6)	0.0543 (8)	0.0413 (6)	−0.0031 (5)	0.0042 (5)	−0.0005 (6)
C5	0.0270 (5)	0.0373 (6)	0.0380 (6)	0.0033 (4)	0.0059 (4)	0.0035 (5)
C6	0.0280 (6)	0.0491 (7)	0.0375 (6)	0.0003 (5)	0.0054 (4)	0.0043 (5)
C7	0.0289 (6)	0.0473 (7)	0.0325 (6)	0.0002 (5)	0.0080 (4)	0.0012 (5)
C8	0.0327 (6)	0.0541 (7)	0.0313 (5)	0.0025 (5)	0.0046 (4)	0.0070 (5)
C9	0.0262 (5)	0.0547 (8)	0.0357 (6)	0.0029 (5)	0.0035 (4)	0.0041 (5)
N1	0.0306 (5)	0.0593 (7)	0.0430 (6)	−0.0050 (5)	0.0044 (4)	0.0050 (5)
O1	0.0272 (4)	0.0811 (7)	0.0401 (5)	0.0027 (4)	0.0090 (3)	0.0173 (5)

### Geometric parameters (Å, °)

**Table:**

C1—N1	1.3388 (16)	C6—O1	1.4192 (16)
C1—C2	1.373 (2)	C6—H6A	0.9700
C1—H1	0.9300	C6—H6B	0.9700
C2—C3	1.374 (2)	C7—O1	1.3754 (15)
C2—H2	0.9300	C7—C8	1.3834 (18)
C3—C4	1.3791 (18)	C7—C9	1.3861 (18)
C3—H3	0.9300	C8—C9^i^	1.3836 (17)
C4—C5	1.3838 (18)	C8—H8	0.9300
C4—H4	0.9300	C9—C8^i^	1.3837 (17)
C5—N1	1.3369 (17)	C9—H9	0.9300
C5—C6	1.5023 (17)		
			
N1—C1—C2	123.93 (12)	O1—C6—H6A	110.2
N1—C1—H1	118.0	C5—C6—H6A	110.2
C2—C1—H1	118.0	O1—C6—H6B	110.2
C1—C2—C3	118.55 (11)	C5—C6—H6B	110.2
C1—C2—H2	120.7	H6A—C6—H6B	108.5
C3—C2—H2	120.7	O1—C7—C8	115.96 (11)
C2—C3—C4	118.62 (12)	O1—C7—C9	124.59 (11)
C2—C3—H3	120.7	C8—C7—C9	119.44 (11)
C4—C3—H3	120.7	C7—C8—C9^i^	121.12 (11)
C3—C4—C5	119.27 (12)	C7—C8—H8	119.4
C3—C4—H4	120.4	C9^i^—C8—H8	119.4
C5—C4—H4	120.4	C8^i^—C9—C7	119.43 (11)
N1—C5—C4	122.58 (11)	C8^i^—C9—H9	120.3
N1—C5—C6	115.82 (11)	C7—C9—H9	120.3
C4—C5—C6	121.58 (11)	C5—N1—C1	117.05 (11)
O1—C6—C5	107.74 (10)	C7—O1—C6	117.84 (9)

Symmetry codes: (i) −*x*+1, −*y*, −*z*+2.

### Hydrogen-bond geometry (Å, °)

**Table:**

*D*—H···*A*	*D*—H	H···*A*	*D*···*A*	*D*—H···*A*
C9—H9···N1^ii^	0.93	2.68	3.587 (2)	165

Symmetry codes: (ii) −*x*+2, −*y*, −*z*+2.
